# Mapping ammonia emission plumes using shortwave infrared imaging spectroscopy

**DOI:** 10.1073/pnas.2605694123

**Published:** 2026-06-12

**Authors:** Nicholas Balasus, Daniel H. Cusworth, Jinsol Kim, Daniel J. Varon, Charles E. Miller, Riley M. Duren

**Affiliations:** ^a^Carbon Mapper, Pasadena, CA 91101; ^b^ Department of Aeronautics and Astronautics, Massachusetts Institute of Technology, Cambridge, MA 02139; ^c^ Jet Propulsion Laboratory, California Institute of Technology, Pasadena, CA 91101

**Keywords:** ammonia, satellites, remote sensing

## Abstract

Atmospheric ammonia emissions are harmful to ecosystems and human health. These emissions have traditionally been monitored using thermal infrared spectrometers, though such techniques are limited by thermal contrast requirements, the coarse spatial resolution of existing satellite sensors, and low measurement frequency of higher-resolution aerial surveys. Here, we show that ammonia emissions can be quantified using shortwave infrared imaging spectroscopy, circumventing these challenges by using reflected sunlight instead of thermal emission for signal and by enabling a large class of existing and future imaging spectrometers to enter the ammonia observing system. As a proof of concept for this capability, we use Tanager-1 satellite data to quantify emissions from industrial point sources of ammonia in Pakistan and Uzbekistan.

Atmospheric ammonia pollutes the environment, both by introducing reactive nitrogen into sensitive ecosystems and by serving as a precursor to hazardous particulate matter (1). Currently, agricultural emissions dominate, such as those from synthetic fertilizers or animal waste, while emissions from the energy sector could grow if ammonia is widely adopted as a fuel or hydrogen carrier (2).

Global satellite observations of atmospheric ammonia have identified shortcomings in our understanding of ammonia emissions, including a large underestimate of emissions from industrial processes (3). These observations rely on thermal infrared spectrometers, taking advantage of the strong ammonia absorption features between 10 to 11 μm, but their applications to emissions monitoring are limited to large and isolated sources due to the coarse spatial resolution (km-scale) of the observations (4). Thermal infrared spectrometers have also been flown on airborne platforms to achieve high spatial resolution, successfully mapping emissions from industrial, agricultural, and natural sources (5–7), though such flights are presently infrequent. Besides these current practical limitations, the requirement for thermal contrast between the surface and atmosphere presents technical challenges, including limiting sensitivity to surface-level emissions and requiring knowledge of plume altitude, the latter of which can greatly complicate emission estimation (6).

Here, we show that ammonia absorption features in the shortwave infrared (8) allow for emission plumes to be mapped using shortwave infrared imaging spectroscopy, where thermal contrast is not required and high-resolution observations from airborne and spaceborne platforms are more common. As a proof of concept, we use observations from the Tanager-1 satellite to retrieve atmospheric ammonia column enhancements at 30 m spatial resolution, finding large emissions from industrial sources in Pakistan and Uzbekistan. The ability to quantify ammonia emissions using shortwave infrared imaging spectroscopy has the potential to significantly expand the ammonia observing system.

## Methods

We use calibrated radiance data from the spaceborne Tanager-1 pushbroom imaging spectrometer which has a spectral range covering 400 to 2,500 nm with 5 nm spectral sampling and 30 m spatial resolution (9). We use the matched filter algorithm to estimate the path length ammonia concentration enhancements $\hat{\alpha }$ from the at-sensor radiance spectrum $x\in R^{n}$ for each ground pixel. Here, n is the number of spectral bands, which we limit to those between 1,400 and 2,500 nm.

[1] $$\begin{array}{c} \hat{\alpha }=\frac{{\left(x-\mu \right)}^{T}\Sigma^{-1}t}{t^{T}\Sigma^{-1}t} \end{array}$$

We take μ to be the mean radiance spectrum for all pixels observed by the same detector in the along-track direction of the sensor and $\Sigma \in R^{n\times n}$ to be the covariance of the same sample. The target signature t is the product of μ and the unit absorption spectrum s, where s describes the relative change in the at-sensor radiance per unit change in ammonia path length enhancement α.

To calculate s, we model the at-sensor radiance spectrum I using a nonscattering radiative transfer model (10) with the US standard atmosphere (11), with the methane and carbon dioxide profiles scaled based on surface concentrations of 1.8 and 420 ppm respectively. Absorption coefficients are from the HITRAN2024 database (12), to which we apply Voigt line shapes using the layer-specific temperature and pressure values from the US standard atmosphere. The high-resolution (0.01 cm^−1^) spectrum I is convolved to the instrument-resolution spectrum L assuming Gaussian spectral responses at each band based on calibrated band widths. We then calculate s as

**Fig. 1.**
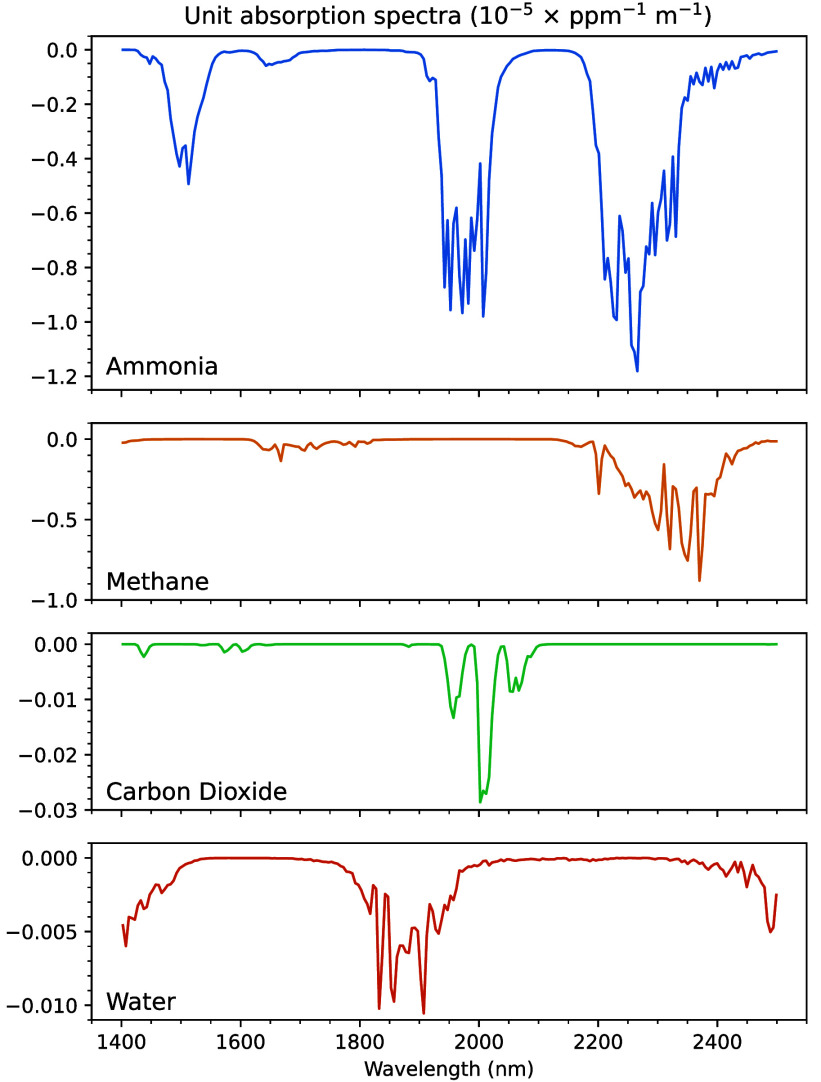
Unit absorption spectra for ammonia, methane, carbon dioxide, and water for 1,400 to 2,500 nm. The spectra depict the relative change in the at-sensor radiance per unit change in a path-length enhancement of the given gas for the Tanager-1 sensor. Values are scaled by $10^{5}$ for visualization.

[2] $$\begin{array}{c} s=\frac{\ln L_{\text{enh}}-\ln L_{\text{bkg}}}{\alpha }, \end{array}$$

where $L_{\text{bkg}}$ is the modeled at-sensor radiance for the standard atmosphere and $L_{\text{enh}}$ is the same but for a surface enhancement of ammonia corresponding to α=2,000 ppm·m (13). Fig. 1 shows unit absorption spectra s for ammonia as well as methane, carbon dioxide, and water.

We convert the path length enhancements $\hat{\alpha }$ (ppm·m) to vertical column mass enhancements ΔΩ (kg m^−2^) using the viewing geometry and US standard atmosphere surface conditions. We delineate the plumes using thresholding (minimum of $10^{18}$ molecules cm^−2^), a 5-pixel window median filter, and a 1-pixel dilation, and then calculate an ammonia emission rate Q (kg h^−1^) using the integrated mass enhancement method (14).

[3] $$\begin{array}{c} Q=\frac{U}{L}\sum_{i}\Delta \Omega_{i}A_{i} \end{array}$$

Here, U (m h^−1^) is the 10 m wind from ECMWF IFS HRES, L (m) is the maximum distance between boundary points of the convex hull of the plume mask, and $A_{i}$ (m^2^) is the area of each pixel i within the delineated plume (9). By using this approach, we assume that the lifetime to chemical and deposition losses are sufficiently long relative to transport that they can be ignored.

To estimate uncertainty, we use an ensemble approach, quantifying the emission rate of each plume 100,000 times. For each iteration, we 1) use a randomly sampled 10 m wind speed from the 9 km grid cell and 15-min time step of ECMWF IFS HRES centered on and adjacent to our observation, and 2) add Gaussian noise uniformly to each pixel in the plume, using a SD of 2.2 × 10^17^ molecules cm^−2^ derived from out-of-plume variability. From the resulting distribution of emissions, we report the mean as our best estimate and the 2.5th and 97.5th percentiles as our uncertainty.

## Results and Discussion

**Fig. 2.**
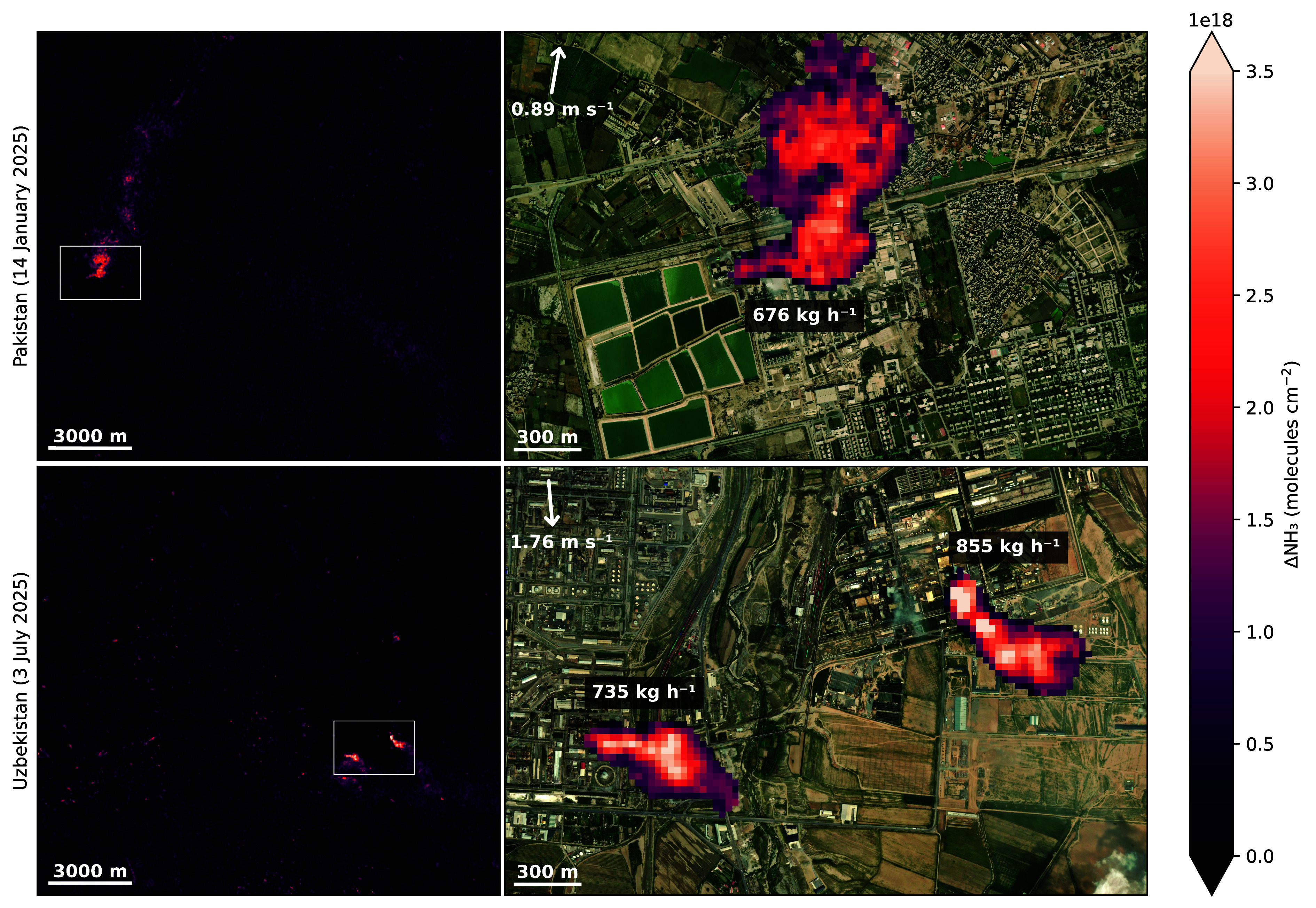
Ammonia emissions from industrial sources in Pakistan (*Top*) and Uzbekistan (*Bottom*) mapped using shortwave infrared radiance data from Tanager-1. On the *Left* is a portion of the full Tanager-1 scene to show background variability, with the white boxes outlining the zoomed-in regions on the *Right* that contain the plumes. The winds and the inferred emission rates are *Inset* and ammonia vertical column enhancements are shown. The background imagery is from Esri World Imagery.

Fig. 2 shows ammonia emission plumes from two industrial fertilizer production plants in Pakistan and Uzbekistan. We infer an emission rate of 676 (438 to 968) kg h^−1^ from the fertilizer production plant in Pakistan. Using data from 2013 to 2017, Dammers et al. (4) estimated emission rates ranging from 3,212 to 8,035 kg h^−1^ for this site depending on the thermal infrared spectrometer used. In Uzbekistan, we quantify two nearby sources with emission rates of 735 (529 to 948) and 855 (629 to 1,086) kg h^−1^ for a total emission rate of 1,590 kg h^−1^. Using data from 2008 to 2016, Van Damme et al. (3) estimated an emission rate of 2,714 kg h^−1^ for the same site. The emission estimates from the coarse resolution thermal infrared sounders that we compare our estimates against (3, 4) are sensitive to the ammonia lifetime assumed or fit, represent long-term averages that do not overlap with our observation times, and may also be sensitive to other nearby sources. Continued sampling by Tanager-1 would allow for more rigorous quantification assessment.

Our ammonia matched filter retrieval uses radiance data from 1,400 to 2,500 nm. To confirm that the ammonia plumes that we have identified are not artifacts due to absorption from other gases shown in Fig. 1 with absorption features in this range, we conduct retrievals over the 1,425 to 1,575, 1,850 to 2,100, and 2,175 to 2,300 nm windows. Although the signal is weak in the first two windows due to water vapor absorption, in all cases, the ammonia plumes remain distinct.

We estimate a minimum detection limit ($Q_{\min}$) for Tanager-1 ammonia observations through mass balance arguments: $Q_{\min}\propto UW\sigma$ (15). Assuming U=3 m s^−1^, with pixel size W=30 m, and precision $\sigma =2.2\times 10^{17}$ molecules cm^−2^ (estimated as the SD of out-of-plume retrieved enhancements), the minimum detection limit under these conditions is 40 kg h^−1^. This analytical approach from Jacob et al. (15) has been empirically validated for Tanager-1 methane observations in Duren et al. (9), though the estimate made here is based only on two scenes and thus may not represent diverse observation conditions and should be interpreted as only an initial estimate.

This study serves as a proof of concept that ammonia emission plumes can be mapped using shortwave infrared imaging spectroscopy, circumventing technical and practical challenges from the current ammonia observing system focused on the thermal infrared spectral range. There is a class of existing airborne and spaceborne sensors (e.g., AVIRIS, EMIT, PRISMA, EnMAP) that cover the ammonia shortwave infrared absorption features, albeit with variable spectral and spatial resolution and coverage. Next-generation instruments with high spectral resolution in the 2,200 to 2,400 nm range aimed at methane absorption features could offer heightened sensitivity to ammonia.

## Data Availability

The full-scene Tanager-1 ammonia vertical column enhancement retrievals are archived on Zenodo at https://doi.org/10.5281/zenodo.19353537 (16).

## References

[1] J. W. Erisman, How ammonia feeds and pollutes the world. Science **374**, 685–686 (2021). 34735256 10.1126/science.abm3492

[2] M. B. Bertagni , Minimizing the impacts of the ammonia economy on the nitrogen cycle and climate. Proc. Natl. Acad. Sci. U.S.A. **120**, e2311728120 (2023). 37931102 10.1073/pnas.2311728120 PMC10655559

[3] M. V. Damme , Industrial and agricultural ammonia point sources exposed. Nature **564**, 99–103 (2018). 30518888 10.1038/s41586-018-0747-1

[4] E. Dammers , NH_3_ emissions from large point sources derived from CrIS and IASI satellite observations. Atmos. Chem. Phys. **19**, 12261–12293 (2019).

[5] L. Kuai , Quantification of ammonia emissions with high spatial resolution thermal infrared observations from the Hyperspectral Thermal Emission Spectrometer (HyTES) airborne instrument. IEEE J. Sel. Top. Appl. Earth Obs. Remote Sens. **12**, 4798–4812 (2019).

[6] L. Noppen , Constraining industrial ammonia emissions using hyperspectral infrared imaging. Remote Sens. Environ. **291**, 113559 (2023).

[7] S. Hasheminassab , Tracing ammonia emission sources in California’s Salton Sea region: Insights from airborne longwave-infrared hyperspectral imaging and ground monitoring. Atmos. Chem. Phys. **25**, 11935–11950 (2025).

[8] P. Cacciani , The ammonia absorption spectrum between 3900 and 6350 cm^−1^: ^15^NH_3_ contribution and a recommended list for natural ammonia. J. Quant. Spectrosc. Radiat. Transf. **329**, 109148 (2024).

[9] R. Duren , The Carbon Mapper emissions monitoring system. Atmos. Meas. Tech. **18**, 6933–6958 (2025).

[10] G. Kuhlmann , Evidence of successful methane mitigation in one of Europe’s most important oil production region. Atmos. Chem. Phys. **25**, 5371–5385 (2025).

[11] G. P. Anderson , AFGL Atmospheric Constituent Profiles (0–120 km) (Air Force Geophysics Laboratory, 1986).

[12] I. E. Gordon , The HITRAN2024 molecular spectroscopic database. J. Quant. Spectrosc. Radiat. Transf. **353**, 109807 (2026).

[13] M. Knapp , Spectrometric imaging of sub-hourly methane emission dynamics from coal mine ventilation. Environ. Res. Lett. **18**, 044030 (2023).

[14] D. J. Varon , Quantifying methane point sources from fine-scale satellite observations of atmospheric methane plumes. Atmos. Meas. Tech. **11**, 5673–5686 (2018).

[15] D. J. Jacob , Satellite observations of atmospheric methane and their value for quantifying methane emissions. Atmos. Chem. Phys. **16**, 14371–14396 (2016).

[16] N. Balasus, Tanager-1 ammonia vertical column enhancements. Zenodo. 10.5281/zenodo.19353537. Deposited 31 March 2026.

